# Paget Disease of the Calvarium: A Case Report

**DOI:** 10.7759/cureus.116917

**Published:** 2026-09-25

**Authors:** Kasra Navabi, Erica Patel

**Affiliations:** 1 Medicine/Endocrinology, University of California Los Angeles David Geffen School of Medicine, Los Angeles, USA

**Keywords:** bone scintigraphy, bone-specific alkaline phosphatase, calvarium, paget disease of bone, temporal bone, tinnitus, zoledronic acid

## Abstract

Paget disease of bone (PDB) is a focal skeletal disorder characterized by excessive and disorganized bone remodeling. When the skull is involved, the disease may also affect the temporal bones. We present the case of a 68-year-old woman referred for evaluation of persistently elevated alkaline phosphatase (ALP), ultimately diagnosed with Paget disease of the calvarium with bilateral temporal bone involvement. The diagnosis was established through a systematic workup spanning nearly four years, including serial biochemical monitoring, exclusion of alternative causes of increased bone turnover, whole-body bone scintigraphy, skull radiographs, and computed tomography of the skull. Total ALP remained persistently elevated, and the bone-specific fraction rose progressively over the course of follow-up while calcium and parathyroid hormone remained within their reference ranges. She was treated with a single intravenous infusion of zoledronic acid. This case highlights the importance of serial monitoring of bone-specific ALP in the evaluation of persistently elevated ALP, the potential for subtle, easily overlooked bone scintigraphy findings in early or evolving Paget disease, and the role of multimodality imaging in defining the extent of calvarial and temporal bone involvement.

## Introduction

Paget disease of bone (PDB) is the second most prevalent metabolic bone disorder, characterized by focal areas of increased and disorganized bone remodeling [1,2]. The disease preferentially targets the axial skeleton, most frequently affecting the pelvis (70%), femur (55%), lumbar spine (53%), skull (42%), and tibia (32%) [3]. PDB is rare before the age of 55 years but increases in prevalence thereafter, affecting approximately 2-3% of individuals over age 55 in the United States [4]. Epidemiological studies have demonstrated a declining incidence in several countries over recent decades, though the reasons remain incompletely understood [5,6].

PDB may be monostotic, affecting a single bone, or polyostotic, involving two or more bones; polyostotic disease is more common, accounting for roughly two-thirds of cases, while monostotic disease, as in the present case, accounts for the remainder and is generally associated with a more limited symptom burden and lower risk of complications [1,2].

The pathogenesis of PDB involves genetic and environmental factors. The most important susceptibility gene is SQSTM1, which encodes p62, a protein involved in the NF-κB signaling pathway. Mutations in SQSTM1 have been identified in 40-50% of familial cases and 5-10% of sporadic cases [7]. Environmental factors, including possible paramyxoviral infections, may also contribute to disease pathogenesis [4].

Many patients with PDB are asymptomatic, and the disease is often discovered incidentally through elevated serum alkaline phosphatase (ALP) or abnormal imaging [1,8]. When the skull is involved, complications may include hearing loss, tinnitus, vestibular dysfunction, cranial nerve palsies, and rarely, basilar invagination [3,4]. Hearing loss is the most common neurological complication of PDB, originally attributed to eighth cranial nerve compression but now believed to result primarily from cochlear damage related to loss of bone mineral density in the otic capsule [3,9,10]. Temporal bone involvement has been reported in approximately two-thirds of patients with skull PDB, with hearing impairment occurring in 30-50% and vestibular dysfunction in 20-25% of these cases [9].

We present a case of Paget disease of the calvarium with bilateral temporal bone involvement, diagnosed through a systematic and prolonged workup of persistently elevated ALP.

## Case presentation

A 68-year-old woman was referred for evaluation of persistently elevated ALP. Her medical history was notable for osteopenia without osteoporosis, identified on bone densitometry performed early in the evaluation period. She was not taking regular dairy products. Her medication regimen included vitamin D 2000 IU daily, calcium 1200 mg daily, and a daily multivitamin.

Biochemical evaluation

Serial laboratory evaluation spanning nearly four years demonstrated persistently elevated total ALP with a progressively rising bone-specific fraction (Table 1). At initial evaluation on Day 1, total ALP was at the upper limit of the reference range, with normal serum calcium, albumin, creatinine, and estimated glomerular filtration rate.

**Table 1:** Serial biochemical parameters over the evaluation period Values outside the reference range are shown in bold. Reference ranges are those of the reporting laboratory and may vary by assay. Bone isoenzyme (%) denotes the bone fraction of total ALP, for which a predominant bone fraction supports a skeletal source rather than a fixed reference interval. Vitamin D reflects 25-hydroxyvitamin D (≥30 ng/mL indicates sufficiency); GFR is expressed in mL/min/1.73 m^2^. ALP: alkaline phosphatase; PTH: parathyroid hormone; GFR: glomerular filtration rate; - not measured or not applicable

Study day	Total ALP (U/L)	Bone-specific ALP (U/L)	Bone isoenzyme (%)	Calcium (mg/dL)	PTH (pg/mL)	Vitamin D (ng/mL)	GFR (mL/min)
Day 1	132	-	-	9.8	-	-	73
Day 315	124	-	-	10.2	-	-	66
Day 441	144	-	-	-	-	-	-
Day 652	167	-	-	10.1	-	33	70
Day 784	139	41.3	74	10.2	41	-	66
Day 862	135	38.1	-	10.0	-	29	63
Day 923	121	26.5	-	10.0	64	34	-
Day 1015	129	29.8	70	10.1	60	-	78
Day 1144	138	33.7	-	10.2	40	39	60
Day 1224	132	-	78	-	-	-	-
Day 1322	153	104.0	-	9.7	-	-	70
Day 1406	147	-	-	10.0	57	47	61
Reference range	37-133	0-55	-	8.5-10.2	15-65	30-100	≥60

Subsequent evaluations confirmed persistently elevated or high-normal total ALP, with isoenzyme fractionation demonstrating a predominant bone origin. Bone-specific ALP rose progressively across successive measurements and was markedly elevated by Day 1322, having increased several-fold over the preceding year (Table 1).

Calcium metabolism remained normal throughout the evaluation period, with serum calcium, corrected ionized calcium, and phosphorus all within their reference ranges. Parathyroid hormone remained within the reference range at every measurement. Serum 25-hydroxyvitamin D was mildly low at the outset and rose into the sufficient range with supplementation. Thyroid function was normal. Serum immunofixation revealed no monoclonal immunoglobulins, effectively excluding multiple myeloma. Renal function was mildly reduced but stable throughout (Table 1).

Imaging studies

An initial whole-body bone scintigraphy performed on Day 1076 was prospectively interpreted as showing no abnormal skeletal uptake. On retrospective review, however, a faint increase in tracer activity was already discernible in the calvarium at that time, representing an early, underrecognized manifestation of the disease. Given the continued elevation and progressive rise in bone-specific ALP, a repeat whole-body bone scan was performed on Day 1395. This study revealed markedly more intense, asymmetric tracer activity in the skull relative to the remainder of the body on both anterior and posterior views, with no focal abnormal uptake elsewhere in the skeleton, confirming monostotic disease limited to the calvarium (Figure 1).

**Figure 1:**
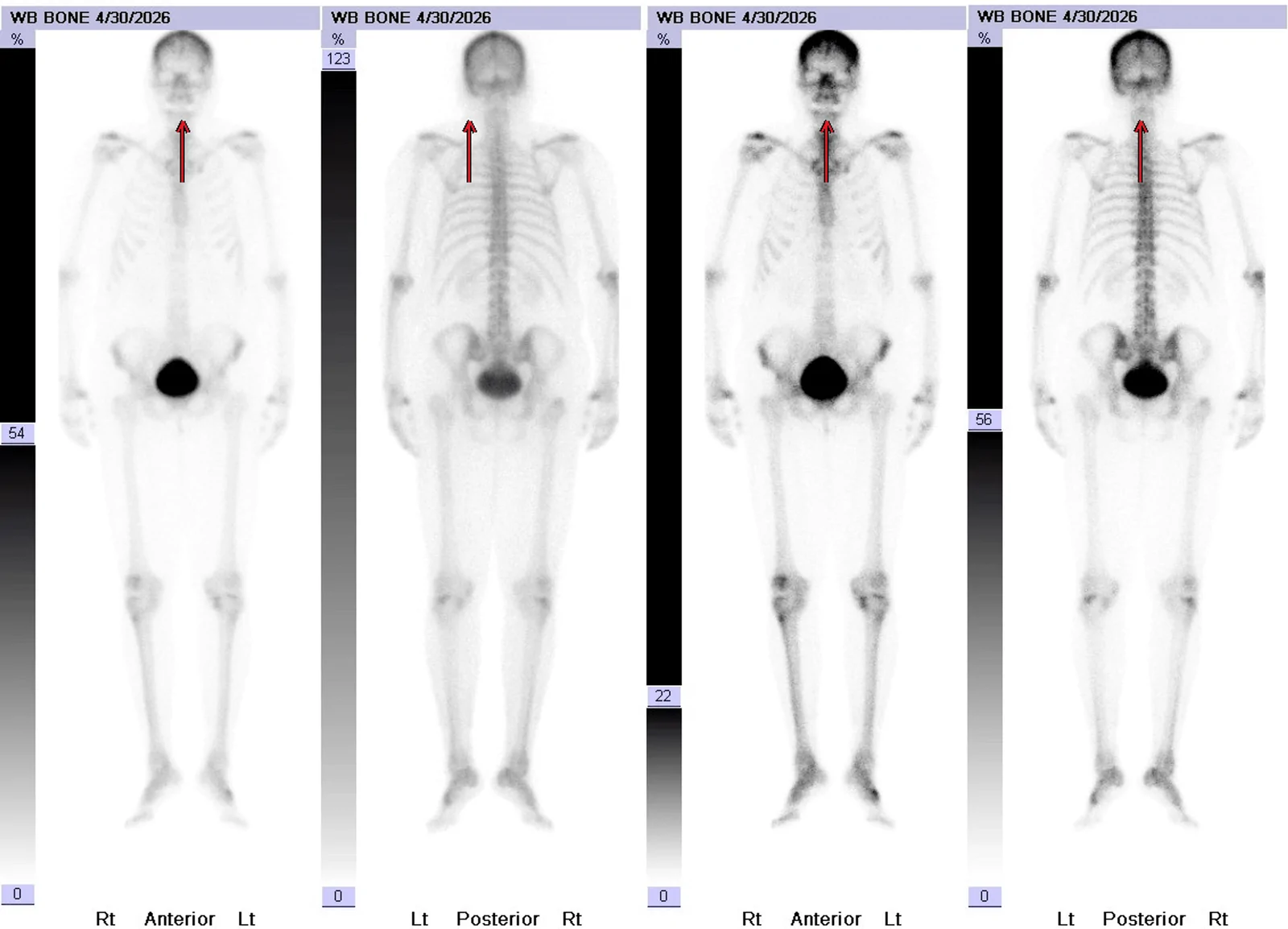
Whole-body technetium-99m methylene diphosphonate bone scintigraphy obtained on Day 1395 Anterior and posterior whole-body images at two different intensity scales demonstrate asymmetrically intense tracer activity in the skull relative to the remainder of the body. No focal abnormal uptake is identified elsewhere in the skeleton, confirming monostotic Paget disease limited to the calvarium. Arrows indicate the abnormally increased tracer uptake within the calvarium, with one arrow placed on each of the four whole-body views.

Skull radiographs were obtained on Day 1403 in three projections. The lateral view (Figure 2A) demonstrated diffuse calvarial thickening with widening of the diploic space and a heterogeneous, mottled "cotton wool" appearance involving the frontal, parietal, and occipital bones, with relative sparing of the skull base and facial bones. The posteroanterior view (Figure 2B) confirmed symmetric, diffuse calvarial thickening with the characteristic mixed sclerotic-lucent pattern extending to the temporal regions bilaterally. The anteroposterior view (Figure 2C) corroborated these findings, demonstrating the diffuse pagetoid involvement of the calvarium.

**Figure 2:**
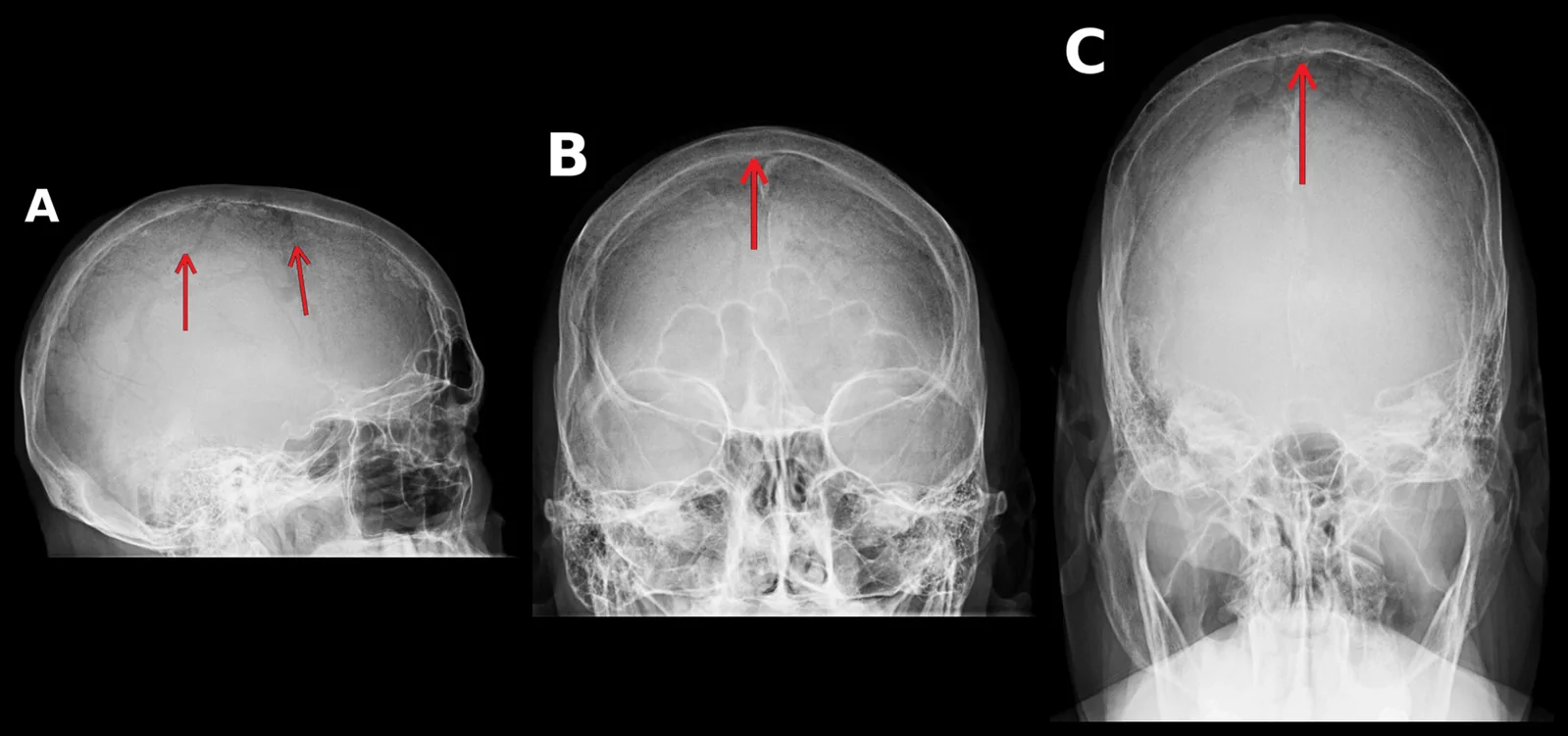
Skull radiographs (A) Lateral view demonstrating diffuse calvarial thickening with widening of the diploic space and a heterogeneous, mottled "cotton wool" appearance involving the frontal, parietal, and occipital bones, with relative sparing of the skull base and facial bones. (B) Posteroanterior view confirming symmetric, diffuse calvarial thickening with the characteristic mixed sclerotic-lucent pattern extending to the temporal regions bilaterally. (C) Anteroposterior view corroborating the diffuse pagetoid involvement of the calvarium. Panel letters A to C denote the three radiographic projections. Arrows indicate representative areas of calvarial pagetic change, marking the thickened diploic space and the mottled sclerotic-lucent bone.

A follow-up computed tomography (CT) scan of the skull was performed on Day 1406. The sagittal CT image (Figure 3A) demonstrated marked thickening of the calvarial diploic space with mixed areas of sclerosis and lucency throughout the frontal, parietal, and occipital regions. The coronal CT image (Figure 3B) confirmed diffuse bilateral calvarial thickening with expansion of the diploic space extending to the squamosal portions of the temporal bones bilaterally. The axial CT image at the vertex level (Figure 3C) showed circumferential calvarial thickening with the characteristic mixed sclerotic-lucent pattern of the diploic space, consistent with the active/mixed phase of Paget disease. The axial CT image at the skull base level (Figure 3D) demonstrated involvement of the temporal bones bilaterally. There were no areas of bone erosion, periosteal reaction, or associated soft tissue mass.

**Figure 3:**
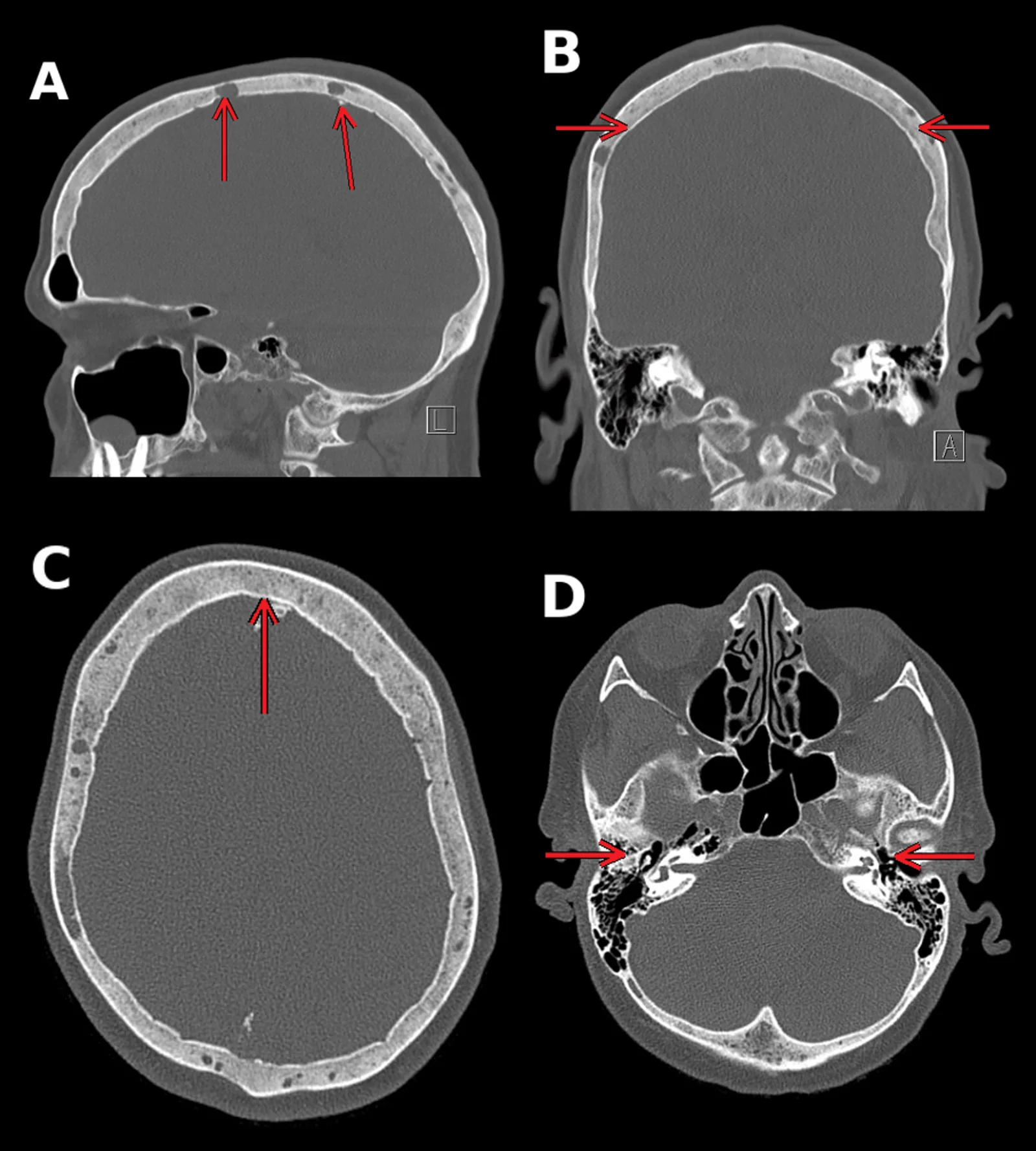
CT of the skull demonstrating Paget disease of the calvarium (A) Sagittal view showing marked thickening of the calvarial diploic space with mixed sclerotic and lucent areas throughout the frontal, parietal, and occipital regions. (B) Coronal view demonstrating diffuse bilateral calvarial thickening with involvement of the squamosal portions of the temporal bones. (C) Axial view at the vertex level showing circumferential calvarial thickening with the characteristic mixed sclerotic-lucent pattern. (D) Axial view at the skull base level demonstrating bilateral temporal bone involvement. Arrows mark the areas of interest in each panel: in panels A to C, they indicate representative regions of diploic space thickening with mixed sclerosis and lucency, and in panel D, they indicate the involved petrous temporal bones bilaterally.

Clinical symptoms

The patient reported two symptoms. First, she described tinnitus present for more than one year. Second, she reported longstanding balance difficulty, which she had initially attributed to a prior diastasis recti surgery affecting her core stability. Following identification of bilateral temporal bone involvement, the possibility that these symptoms were related to Paget disease was considered.

Physical examination

Vital signs were within normal limits: blood pressure 119/73 mmHg, pulse 65 beats per minute, temperature 36.4°C, oxygen saturation 98%, weight 83.8 kg, and height 1.76 m. On balance testing performed at a prior executive physical examination, she was unable to maintain a single-leg stance with eyes closed for even two seconds, a finding below what is expected for her age.

Diagnosis

Based on the convergence of biochemical, scintigraphic, radiographic, and CT findings, a diagnosis of Paget disease of the calvarium with bilateral temporal bone involvement was established.

Management

The patient was initiated on treatment with a single intravenous infusion of zoledronic acid 5 mg, administered once over 15 minutes, with adequate pre-infusion hydration. Vitamin D and calcium supplementation were continued. The patient was counseled regarding the acute-phase reaction (fever, myalgias, arthralgias) that occurs in approximately 30% of treatment-naïve patients [11].

The patient was referred to otolaryngology and, at the time of this report, was awaiting consultation. Further evaluation, including audiometry, vestibular function testing, and any dedicated temporal bone imaging, will be determined by the otolaryngology team. Follow-up biochemical monitoring with total ALP and bone-specific ALP was planned at three to six months post-infusion to assess treatment response.

## Discussion

This case illustrates several instructive features of PDB involving the skull. Three aspects are of particular teaching value: the serial rise in bone-specific ALP that ultimately established the diagnosis; the subtle early bone scintigraphy findings that were prospectively overlooked and became unequivocal only on later imaging; and the bilateral temporal bone involvement, a site at risk for otologic complications.

Diagnostic challenges and the value of serial monitoring

The diagnosis of PDB in this patient was established through a systematic workup spanning nearly four years. The initial presentation was an incidentally discovered, persistently elevated ALP - the most common initial finding in PDB [1,8]. Isoenzyme fractionation confirmed the bone origin of the elevated ALP, and the several-fold rise in bone-specific ALP over the final year of follow-up reflected increasing metabolic activity at the pagetic site (Table 1).

Notably, the initial whole-body bone scintigraphy on Day 1076 was prospectively interpreted as negative, yet retrospective review revealed that faint calvarial uptake was already present; the repeat scan approximately 10 months later demonstrated markedly more intense skull uptake (Figure 1). This temporal evolution is consistent with the known slow progression of Paget disease [8]. Radionuclide bone scintigraphy is considered the most sensitive imaging modality for detecting PDB, but early lesions may produce only subtle uptake that is easily overlooked on initial interpretation, and sclerotic ("burned out") lesions may appear normal [11,12]. This case underscores the importance of careful re-review of prior imaging and repeat scintigraphy when clinical suspicion remains high despite an initially unremarkable bone scan.

The skull radiographs (Figure 2) demonstrated the classic "cotton wool" appearance - a pathognomonic finding in Paget disease of the skull that results from the coalescence of multiple foci of sclerotic bone within the thickened calvarium [1]. The CT findings of diffuse calvarial thickening with mixed sclerosis and lucency (Figure 3), without bone erosion, periosteal reaction, or soft tissue mass, are classic for the mixed (active) phase of PDB and effectively exclude malignant transformation [1]. The Endocrine Society Clinical Practice Guideline recommends that the diagnosis of PDB be confirmed by characteristic radiographic findings, with radionuclide bone scintigraphy used to define the full extent of metabolically active disease [3,12].

The bone scintigraphy (Figure 1) also confirmed that disease was limited to the calvarium (monostotic), which generally carries a lower risk of complications such as pathological fracture or secondary osteoarthritis than polyostotic disease [4,8].

Temporal bone involvement and otologic manifestations

The bilateral temporal bone involvement in this patient is of particular clinical significance. The skull and temporal bones become involved in approximately two-thirds of patients with skull PDB [9]. In the largest single-institution series of 27 patients with Paget disease of the temporal bone (PDTB), Deep et al. reported that symptoms at presentation included hearing loss (85%), headache (67%), dizziness (52%), and tinnitus (19%) [13]. Of ears with audiometric data, 65% exhibited sensorineural hearing loss and 35% mixed hearing loss [13].

A recent large national database study by Rosen et al., analyzing 8,790 patients with Paget disease, confirmed significantly higher rates of sensorineural hearing loss (9.1% vs. 6.1%, P < 0.001), conductive hearing loss (0.8% vs. 0.46%, P < 0.001), mixed hearing loss (1.45% vs. 0.9%, P = 0.006), tinnitus (4.6% vs. 2.8%, P < 0.001), and vertigo (3.9% vs. 2.8%, P < 0.001) compared with propensity-matched controls [14].

The pathophysiology of hearing loss in PDB has evolved from the original hypothesis of eighth cranial nerve compression to a cochlear mechanism. Monsell demonstrated a strong correlation between cochlear capsule bone mineral density and hearing thresholds in pagetic subjects, supporting a general cochlear mechanism of hearing loss closely related to loss of bone mineral density in the otic capsule [15]. Amilibia et al. confirmed that otic capsule bone density measured in Hounsfield units was significantly decreased in PDTB patients compared with controls, and that this decrease was associated with both air-conduction and bone-conduction hearing thresholds [16]. Histopathological studies have identified multiple mechanisms including stapes footplate fixation or fracture, obliteration of Cotugno’s canal by pagetoid bone remodeling, microfissures of the otic capsule, strial atrophy, and cystic degeneration of the spiral ligament [1,10,17,18].

Our patient’s tinnitus and balance disturbance may be related to the temporal bone involvement by Paget disease, and the bilateral temporal bone involvement demonstrated on CT (Figure 3) provides a plausible anatomical substrate for these symptoms. Vestibular dysfunction has been reported in 20-25% of patients with temporal bone PDB [9]. Nevertheless, because the patient is awaiting otolaryngology consultation and audiometric, vestibular, and dedicated temporal bone imaging studies have not been obtained, a causal relationship cannot be established, and alternative otologic causes remain possible.

Treatment with zoledronic acid

Bisphosphonates are the treatment of choice for PDB, and among available agents, intravenous zoledronic acid is considered the most effective [3,4]. In the pivotal randomized, double-blind trial by Reid et al., a single 5 mg infusion of zoledronic acid achieved a therapeutic response in 96% of patients compared with 74% for risedronate (P < 0.001), with normalization of ALP in 89% versus 58% (P < 0.001) [11]. In long-term open follow-up of responders from that trial, remission was sustained in approximately 87% of zoledronic acid-treated patients, with loss of therapeutic response in only 12.5% over follow-up of up to 6.5 years [19].

Treatment was indicated in this patient given the active disease (rising bone-specific ALP, Table 1) at a site susceptible to progressive complications, namely the temporal bones. The Endocrine Society guideline recommends treatment for patients with active disease at sites where complications could occur, including the skull base near the eighth cranial nerve [3].

Regarding the effect of bisphosphonate therapy on hearing loss, evidence remains limited. The Endocrine Society guideline notes that there are no randomized controlled trials assessing the effect of antipagetic therapy on hearing loss [3]. Donáth et al. reported that bisphosphonate treatment in 25 patients with skull PDB resulted in decreased serum ALP and improved scintigraphic parameters, but audiometric examination did not show significant changes at one year [20]. Generally, treated patients do not appear to have further rapid deterioration of hearing, but hearing loss is largely not reversible [3]. Early identification and vigorous treatment with potent bisphosphonates have been advocated to limit the development and progression of pagetic hearing loss [15].

Differential diagnostic considerations

The workup appropriately excluded several important differential diagnoses. Serum immunofixation was negative for monoclonal immunoglobulins, excluding multiple myeloma [8]. The CT findings showed no bone erosion, periosteal reaction, or soft tissue mass (Figure 3), effectively ruling out sarcomatous transformation, which occurs in less than 0.5% of PDB cases [4,8]. Normal thyroid function excluded hyperthyroidism as a cause of elevated bone turnover. Parathyroid hormone (PTH) levels remained within the reference range with adequate vitamin D repletion, making primary hyperparathyroidism unlikely, though concurrent primary hyperparathyroidism has been reported in patients with PDB and should be monitored [4].

Importance of multidisciplinary management

Finally, this case underscores the value of multidisciplinary management. When temporal bone involvement is identified, referral to otolaryngology for formal audiometric and vestibular evaluation is important to characterize any hearing or vestibular impairment, establish a baseline for monitoring, and guide interventions where indicated [4,16].

This report has limitations. Formal audiometric testing, vestibular testing, and dedicated temporal bone imaging had not been performed at the time of writing, as the patient was awaiting otolaryngology consultation. Consequently, the relationship between the temporal bone involvement and the patient’s otologic symptoms remains unconfirmed, and these findings should be interpreted accordingly.

## Conclusions

This case demonstrates the diagnostic course of a patient with Paget disease of the calvarium and bilateral temporal bone involvement, identified through persistently elevated and progressively rising bone-specific ALP. Key learning points include the importance of serial biochemical monitoring and repeat imaging when clinical suspicion for Paget disease persists despite subtle or initially underrecognized bone scintigraphy findings; the role of multimodality imaging, including skull radiographs, CT, and bone scintigraphy, in confirming the diagnosis and defining the extent of calvarial and temporal bone involvement; and the importance of timely treatment with zoledronic acid. Ongoing monitoring of biochemical markers will be essential to assess treatment response and detect disease progression.
